# Meta-Analysis of MicroRNA-146a rs2910164 G>C Polymorphism Association with Autoimmune Diseases Susceptibility, an Update Based on 24 Studies

**DOI:** 10.1371/journal.pone.0121918

**Published:** 2015-04-01

**Authors:** Changzheng Li, Weijun Fu, Yu Zhang, Liang Zhou, Zhi Mao, Weiran Lv, Juan Li, Ye Zhou

**Affiliations:** 1 Nanfang Hospital, Southern Medical University, Guangzhou, Guangdong, China; 2 School of Traditional Chinese Medicine, Southern Medical University, Guangzhou, Guangdong, China; 3 Department of Medicine, Virginia Commonwealth University, Richmond, Virginia, United States of America; 4 Department of Intensive Care Unit, Chinese PLA General Hospital, Beijing, China; 5 School of Biotechnology, Southern Medical University, Guangzhou, Guangdong, China; VCU, UNITED STATES

## Abstract

**Background:**

Published data showed that the susceptibility of autoimmune diseases (ADs) was associated with the polymorphism rs2910164 in microRNA-146a (miR-146a). However, the results remain controversial so far. Two meta-analyses published in 2013 and 2014 came to opposite conclusions. In order to derive a more precise estimation of the relationship, we performed this meta-analysis.

**Methods:**

We searched the PubMed, OvidSP and CNKI databases (published prior to September 8th, 2014) and extracted data from eligible studies. The procedure of meta-analysis was performed by using the Stata 12.0 software. Random effect model or fixed effect model were chosen respectively, according to the between study heterogeneities.

**Results:**

A total of 24 case-control studies, 11 more than previous meta-analysis on this topic, were involved. We took stratified analyses by different ethnicities and different types of diseases in different genetic models. In Caucasian subgroup, significant increased risks of GC genotype and GC+CC genotype with ADs susceptibility were found in heterozygote model (GC vs GG, OR = 1.38, 95% CI 1.04–1.83, *p* = 0.024) and dominant model (GC+CC vs GG, OR = 1.37, 95% CI 1.01–1.85, *p* = 0.041), respectively. Meanwhile, in other disease subgroup, significant increased risks of C allele, CC genotype and GC+CC genotype were found in allele model (C vs G, OR = 1.16, 95% CI 1.04–1.31, *p* = 0.010), homozygote model (CC vs GG, OR = 1.42, 95% CI 1.10–1.84, *p* = 0.006) and dominant model (GC+CC vs GG, OR = 1.25, 95% CI 1.04–1.51, *p* = 0.020), respectively.

**Conclusions:**

MiR-146a rs2910164 G>C polymorphism was associated with the susceptibility of ADs.

## Introduction

Autoimmune diseases (ADs) are initiated by abnormal immune response to self-antigen, and then come the results including immune-mediated tissue destruction and chronic disabilities [1]. There are more than 100 diseases and syndromes in ADs, which cause a heavy economic burden in the world, about more than $100 billion annually [2]. More and more evidence indicated that genetic backgrounds play an important role in the pathogenesis of ADs, which may be controlled by a common set of susceptibility genes [3, 4].

MicroRNAs (miRNAs) are non-coding single-stranded RNA molecules. By binding to 3’ un-translated regions (UTRs) of targeted messenger RNAs, the miRNAs can repress, degrade or silence the gene expression [5, 6]. Therefore, miRNAs have been demonstrated to affect various functions in both innate and adaptive immune response [7, 8]. Among them, the microRNA-146a (miR-146a) was reported to be involved in both innate and adaptive immunity, especially played an important role in autoimmune disease [8, 9].

Single nucleotide polymorphisms (SNPs) or mutations in miRNAs may alter the expression level of the gene and the susceptibility of some diseases [10–13]. Many studies on the relationship between miR-146a rs2910164 G>C polymorphism and susceptibility of ADs have been performed so far [14–34]. However, the results remain inconsistent. Moreover, two meta-analyses on this issue published in 2013 and 2014 also generated opposite conclusions [35, 36]. Based on the new case-control studies [27, 28, 30–34], we conducted this updated meta-analysis according the criteria PRISMA statement [37], in order to clarify the association between miR-146a rs2910164 G>C polymorphism and susceptibility of ADs.

## Materials and Methods

### Publication search

A systematic search was performed in PubMed, OvidSP and Chinese National Knowledge Infrastructure (CNKI) databases covering all papers published prior to September 8^th^, 2014. The searching strategy was as follow: (autoimmune OR autoimmune disease OR autoimmunity) AND (polymorphism OR polymorphisms OR variation OR variations OR mutation OR mutations OR variant OR variants) AND (Has-mir-146a OR miR146a OR microRNA-146a OR miR-146 OR miR-146a OR rs2910164). The references in the studies were also read to find additional publications on the topic. Articles included should meet the criteria below: (1) case-control study; (2) evaluation of miR-146a rs2910164 G>C polymorphism and risk of ADs; (3) available and usable data of genotype frequency. The articles were excluded if they meet the exclusion criteria below: (1) a case report, review or descriptive study; (2) a lack of normal population as controls; (3) not show the evaluation of miR-146a rs2910164; (4) duplicate data in the studies.

### Date extraction

Two authors (CL and WF) independently extracted the data from eligible studies. Different data extracted by CL and WF were checked by the third author YZ. The remained disagreements were discussed and judged by these three authors. The following information was extracted: the first author, publication year, diseases, country, ethnicity, genotyping methods, number of cases and controls, the gender distribution of cases and controls, number of genotypes and alleles, Hardy-Weinberg equilibrium (HWE) in control subjects, frequency of G allele in controls. Ethnicities were categorized as Caucasian, East Asian, Latin-American and Mediterranean. Study qualities were judged according to the criteria modified from previous publications [38–40] (See S1 Table. “Scale for methodological quality assessment”).

### Statistical analysis

Odds ratios (ORs) and 95% confidence intervals (CIs) were calculated as a measure of the association between miR-146a rs2910164 G>C polymorphism and ADs risk. The widely reported genetic models, allele model, heterozygote model, homozygote model, dominant model and recessive model, were used. In addition to comparing the pooled effects among all subjects, the stratified comparisons were also conducted according to different ethnicities and different diseases. In order to evaluate the accumulation of evidence, the cumulative meta-analysis was performed. The between study heterogeneity was measured by Cochran’s (Q) and Higgins’s (I^2^) tests. If the heterogeneity was considered significant (*p*<0.05), the random effect model was used to estimate the pooled OR. Otherwise, the fixed effect model was conducted. Also, logistic meta-regression analysis was carried out, if there was obvious significant heterogeneity, to explore potential sources of heterogeneity. The examined characteristics include: publication years, countries, genotyping methods, number of alleles and genotypes, number of female and male in cases, and the frequency of G allele in controls. The HWE was examined using Chi-square test with significance set at *p*<0.05. Sensitivity analysis was performed to evaluate the effect of each study on the combined ORs by deleting each study in each time, and to evaluate the effect of studies with low quality or without HWE on the pooled ORs by deleting these studies. Potential publication bias was determined by using Funnel plots and Begg’s test. An asymmetric plot and the *p* value less than 0.05 was recognized as significance. All statistical analyses were performed by Stata 12.0 software (Stata Corp., College Station, TX).

## Results

### Study Characteristics

**Fig 1:**
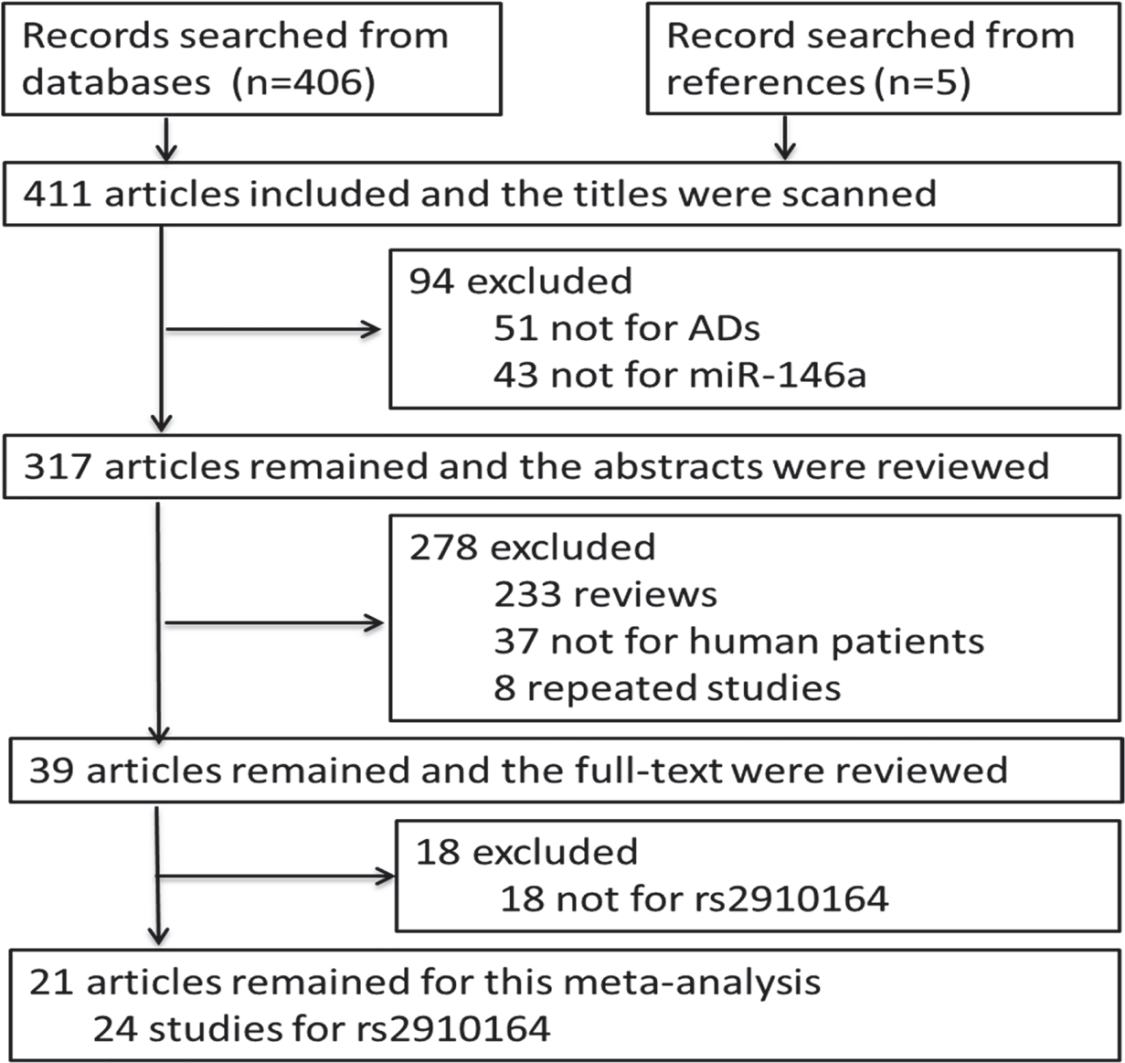
Flowchart for identification of studies included in the meta-analysis. In 411 articles, 51 were found not related to ADs and 43 were found not related to miR-146a by scanning the titles. After that, 233 articles were recognized as reviews, 37 were found not related to human patients and 8 articles were repeated papers by reviewing the abstracts. The full-texts of the left 39 articles were carefully reviewed, in which 18 articles were found not about rs2910164. At last, 21 articles were remained for this meta-analysis, which included 24 case-control studies for rs2910164.

There were 406 articles matching the searching strategy, and additional 5 articles [20, 23, 25, 28, 31] were included by scanning the references of original papers. After step by step of screening the titles, abstracts and full-texts of the articles, as shown in Fig 1, 21 articles were recognized appropriate for this meta-analysis, including 24 studies for rs2910164, 11 studies more than the previous meta-analysis published in 2014 [36].

Within the 21 articles, five kinds of genotyping methods were used. There were 4 racial included, Caucasian, East Asian, Latin-American and Mediterranean. According the different types of diseases, these studies were divided into five subgroups. The studies on Rheumatoid Arthritis (RA), Psoriatic Arthritis (PsA), Juvenile RA (JRA) and Juvenile Idiopathic Arthritis-Enthesitis-Related Arthritis (JIA-ERA) were included into Inflammatory Arthritis (IA) subgroup. The studies on Ulcerative Colitis (UC) and Crohn’s Disease (CD) were included into Inflammatory Bowel Disease (IBD) subgroup. Among these studies, the patients with Behcet’s Disease (BD), Vogt-Koyanagi-Harada syndrome (VKH), Fuchs Uveitis Syndrome (FUS) and Pediatric Uveitis (PU) were all suffering uveitis, which was a common syndrome of ADs. So the Uveitis subgroup was formed. The remained studies, except that for Systemic Lupus Erythematosus (SLE), were included in other diseases subgroup. There were 5 studies not in HWE in control groups (*p*<0.05), but the *p* value of HWE were not less than 0.01. And there was not enough data in another study to generate the HWE in control. The detail characteristics are shown in Table 1.

**Table 1:** Characteristics of published studies of rs2910164.

First author	Year	Diseases	Country	Ethnicity	Sample size		Female/Male		Genotyping methods	Case					Control					HWE of control (p value)	Frequency of G Allele in controls	Quality
										Genotype			Allele		Genotype			Allele				
					case	control	case	control		GG	GC	CC	G	C	GG	GC	CC	G	C			
Chatzikyriakidou	2010a	RA	Greece	Caucasian	136	147	109/27	115/32	PCR-RFLP	73	53	10	199	73	80	53	14	213	81	Y(0.240)	0.72	7
Chatzikyriakidou	2010b	PsA	Greece	Caucasian	29	66	10/19	30/36	PCR-RFLP	14	12	3	40	18	39	18	9	96	36	N(0.011)	0.73	5
Luo	2011	SLE	China	East Asian	816	1080			Direct sequencing				669	963				864	1296		0.40	8
Zhang	2011	SLE	China	East Asian	213	209	201/12	195/14	PCR-RFLP	33	101	79	167	259	23	104	82	150	268	Y(0.239)	0.36	8
Yang	2011	RA	China	East Asian	208	240	172/36	195/45	PCR-RFLP	28	95	85	151	265	30	116	94	176	304	Y(0.529)	0.37	8
Fenoglio	2011	MS	Italy	Caucasian	346	339	240/106	207/132	TaqMan	181	135	30	497	195	195	115	29	505	173	N(0.048)	0.74	7
Okubo	2011	UC	Japan	East Asian	170	403	74/96	208/195	PCR-RFLP	28	67	75	123	217	74	178	151	326	480	Y(0.095)	0.40	7
Lofgren	2012	SLE	Sweden	Caucasian	1109	1428			TaqMan	623	422	64	1668	550	819	531	78	2169	687	Y(0.503)	0.76	7
Jimenez-Morales	2012	SLE	Mexico	Latin-American	367	531	304/63	299/232	TaqMan	163	167	37	493	241	236	229	66	701	361	Y(0.369)	0.66	8
Jimenez-Morales	2012	JRA	Mexico	Latin-American	210	531	125/85	299/232	TaqMan	102	80	28	284	136	236	229	66	701	361	Y(0.369)	0.66	8
Qian	2012	RA	China	East Asian	123	220	105/18	196/24	PCR-LDR	16	65	42	97	149	35	109	76	179	261	Y(0.694)	0.41	8
Sakoguchi	2012	SSc	Japan	East Asian	52	107			PCR-RFLP	1	28	23	30	74	3	53	51	59	155	N(0.013)	0.28	1
Zhou	2012	FUS	China	East Asian	219	612	102/117	262/350	PCR-RFLP	36	91	92	163	275	79	279	254	437	787	Y(0.862)	0.36	9
Hashemi	2013	RA	Iran	Mediterranean	104	110	91/13	70/40	T-ARMS-PCR	57	39	8	153	55	64	37	9	165	55	Y(0.280)	0.75	8
EI-Shal	2013	RA	Egypt	Mediterranean	217	245	217/0	245/0	PCR-RFLP	30	103	84	163	271	15	119	111	149	341	N(0.021)	0.30	7
Gazouli	2013	UC	Greece	Caucasian	210	300	109/101	159/141	PCR-RFLP	126	78	6	330	90	200	90	10	490	110	Y(0.974)	0.82	7
Gazouli	2013	CD	Greece	Caucasian	242	300	134/108	159/141	PCR-RFLP	105	113	24	323	161	200	90	10	490	110	Y(0.974)	0.82	7
Zhou	2014	BD	China	East Asian	809	1132	131/678	513/619	PCR-RFLP	131	440	238	702	916	154	518	460	826	1438	Y(0.670)	0.36	10
Zhou	2014	VKH	China	East Asian	613	1132	287/326	513/619	PCR-RFLP	64	273	276	401	825	154	518	460	826	1438	Y(0.670)	0.36	10
Singh	2014	JIA-ERA	India	Mediterranean	150	216	17/133	15/201	PCR-RFLP	75	56	19	206	94	112	91	13	315	117	Y(0.327)	0.73	8
Lin	2014	IgAN	China	East Asian	404	711	169/235	261/450	PCR-LDR	103	195	106	401	407	219	360	132	798	624	Y(0.454)	0.56	8
Zhao	2014	ITP	China	East Asian	280	270	181/99	156/114	PCR-RFLP	35	134	111	204	356	36	135	99	207	333	Y(0.344)	0.38	8
Wei	2014	PU	China	East Asian	520	1204	278/242	659/545	PCR-RFLP	113	248	159	474	566	163	553	488	879	1529	Y(0.750)	0.37	9
Okada	2014	PM/DM	Japan	East Asian	44	107	28/16		PCR-RFLP	0	29	15	29	59	3	53	51	59	155	N(0.013)	0.28	3

Abbreviations: RA, Rheumatoid Arthritis; PsA, Psoriatic Arthritis; SLE, Systemic Lupus Erythematosus; MS, Multiple Sclerosis; UC, Ulcerative Colitis; JRA, Juvenile Rheumatoid Arthritis; SSc, Systemic Sclerosis; FUS, Fuchs Uveitis Syndrome; CD, Crohn’s Disease; BD, Behcet’s Disease; VKH, Vogt-Koyanagi-Harada syndrome; JIA-ERA, Juvenile Idiopathic Arthritis-Enthesitis-Related Arthritis; IgAN, Immunoglobulin A nephropathy; ITP, Immune Thrombocytopenia; PU, Pediatric Uveitis; PM/DM, Polymyositis/Dermatomyositis.

### Association between miR-146a rs2910164 G>C polymorphism and ADs risk

**Fig 2:**
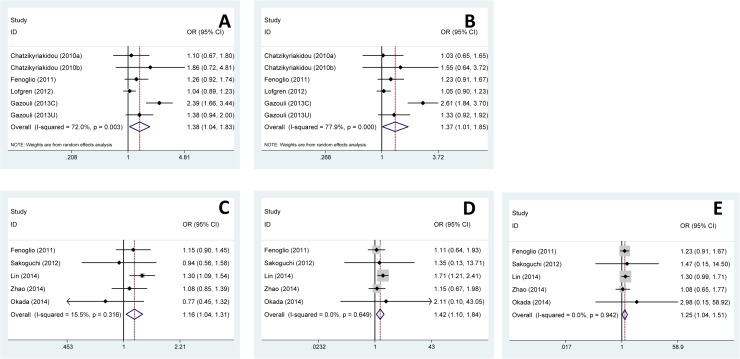
Forest plots of ADs risk associated with rs2910164. **(A-B)** Forest plots of ADs risk associated with rs2910164 stratified analyzed by ethnicities. **(A)** Heterozygote model, GC vs GG, Caucasian subgroup, random model. **(B)** Dominant model, GC+CC vs GG, Caucasian subgroup, random model. **(C-E)** Forest plots of ADs risk associated with rs2910164 stratified analyzed by diseases. **(C)** Allele model, C vs G, Other diseases subgroup, fixed model. **(D)** Homozygote model, CC vs GG, Other diseases subgroup, fixed model. **(E)** Dominant model, GC+CC vs GG, Other diseases subgroup, fixed model. OR: odds ratio; 95% CI: 95% confidence interval.

First, we investigated the overall association between rs2910164 and susceptibility of ADs. No significant difference of the pooled OR was found in any genetic model. However, in terms of stratified analysis, the significance emerged. In Caucasian subgroup, significant increased risks of GC genotype and GC+CC genotype with ADs susceptibility were found in heterozygote model (GC vs GG, OR = 1.38, 95% CI 1.04–1.83, *p* = 0.024) and dominant model (GC+CC vs GG, OR = 1.37, 95% CI 1.01–1.85, *p* = 0.041), respectively (Table 2, Fig 2A and B).

**Table 2:** Stratified analysis of association between ADs risk and rs2910164.

Stratify		Study(n)	Allele model (C vs G)					Heterozygote model (GC vs GG)					Homozygote model (CC vs GG)					Dominant model (GC+CC vs GG)					Recessive model (CC versus GG+GC)				
			Effects size		Heterogeneity		Effect model	Effects size		Heterogeneity		Effect model	Effects size		Heterogeneity		Effect model	Effects size		Heterogeneity		Effect model	Effects size		Heterogeneity		Effect model
			OR	*p*	*I* ^*2*^ *(%)*	*p*		OR	*p*	*I* ^*2*^ *(%)*	*p*		OR	*p*	*I* ^*2*^ *(%)*	*p*		OR	*p*	*I* ^*2*^ *(%)*	*p*		OR	*p*	*I* ^*2*^ *(%)*	*p*	
			(95% CI)		(95% CI)			(95% CI)					(95% CI)					(95% CI)									
Total		24	1.02	0.653	77.9	0.000	Random	1.04	0.556	58.6	0.000	Random	1.01	0.914	73.1	0.000	Random	1.04	0.622	70.7	0.000	Random	1.00	0.995	70.2	0.000	Random
			(0.93–1.13)					(0.91–1.19)					(0.81–1.27)					(0.89–1.21)					(0.85–1.17)				
Ethnicities	Caucasian	6	1.25	0.083	79.5	0.000	Random	**1.38**	**0.024**	72.0	0.003	Random	1.28	0.306	61.9	0.022	Random	**1.37**	**0.041**	77.9	0.000	Random	1.14	0.511	44.7	0.107	Random
			(0.97–1.60)					**(1.04–1.83)**					(0.80–2.07)					**(1.01–1.85)**					(0.77–1.67)				
	East Asian	13	0.97	0.663	79.3	0.000	Random	0.95	0.418	34.0	0.118	Fixed	0.96	0.817	78.8	0.000	Random	0.95	0.642	64.3	0.001	Random	0.96	0.731	80.0	0.000	Random
			(0.85–1.10)					(0.84–1.07)					(0.71–1.32)					(0.77–1.18)					(0.78–1.19)				
	Latin-American	2	0.94	0.440	0.0	0.897	Fixed	0.95	0.633	27.7	0.240	Fixed	0.88	0.466	0.0	0.579	Fixed	0.93	0.519	0.0	0.432	Fixed	0.91	0.550	0.0	0.331	Fixed
			(0.81–1.10)					(0.76–1.18)					(0.63–1.23)					(0.76–1.15)					(0.66–1.25)				
	Mediterranean	3	0.97	0.874	68.9	0.040	Random	0.81	0.417	61.9	0.072	Random	0.93	0.892	82.4	0.003	Random	0.83	0.519	72.4	0.027	Random	1.14	0.725	70.0	0.036	Random
			(0.69–1.38)					(0.48–1.36)					(0.30–2.82)					(0.46–1.48)					(0.56–2.32)				
Diseases	IA	8	0.97	0.607	77.9	0.384	Fixed	0.92	0.384	28.2	0.204	Fixed	0.95	0.767	42.8	0.093	Random	0.94	0.496	29.8	0.190	Fixed	0.99	0.906	8.2	0.366	Fixed
			(0.87–1.09)					(0.77–1.11)					(0.67–1.35)					(0.79–1.12)					(0.82–1.19)				
	SLE	4	0.98	0.625	79.5	0.622	Fixed	1.02	0.748	0.0	0.379	Fixed	0.91	0.468	5.2	0.348	Fixed	1.01	0.857	6.9	0.342	Fixed	0.93	0.544	0.0	0.567	Fixed
			(0.90–1.06)					(0.89–1.17)					(0.71–1.17)					(0.89–1.16)					(0.75–1.16)				
	IBD	3	1.48	0.057	83.5	0.002	Random	1.52	0.096	76.6	0.014	Random	1.82	0.190	76.3	0.015	Random	1.61	0.069	80.3	0.006	Random	1.57	0.178	63.3	0.066	Random
			(0.99–2.21)					(0.93–2.49)					(0.74–4.46)					(0.96–2.68)					(0.82–3.02)				
	Uveitis	4	0.87	0.271	90.5	0.000	Random	0.88	0.429	72.7	0.012	Random	0.75	0.262	88.8	0.000	Random	0.83	0.310	83.1	0.000	Random	0.83	0.293	89.8	0.000	Random
			(0.67–1.12)					(0.65–1.20)					(0.46–1.24)					(0.57–1.20)					(0.58–1.18)				
	Others	5	**1.16**	**0.010**	15.5	0.316	Fixed	1.19	0.092	0.0	0.884	Fixed	**1.42**	**0.006**	0.0	0.649	Fixed	**1.25**	**0.020**	0.0	0.942	Fixed	1.08	0.611	53.1	0.074	Random
			**(1.04–1.31)**					(0.97–1.44)					**(1.10–1.84)**					**(1.04–1.51)**					(0.80–1.47)				

Meanwhile, in other disease subgroup, significant increased risks of C allele, CC genotype and GC+CC genotype with ADs were found in allele model (C vs G, OR = 1.16, 95% CI 1.04–1.31, *p* = 0.010), homozygote model (CC vs GG, OR = 1.42, 95% CI 1.10–1.84, *p* = 0.006) and dominant model (GC+CC vs GG, OR = 1.25, 95% CI 1.04–1.51, *p* = 0.020), respectively (Table 2, Fig 2C, D, E). There was not any significance in IA, SLE, IBD and Uveitis subgroup. However, a trend of increased disease risk of C allele could be found in allele model (C vs G, OR = 1.48, 95% CI 0.99–2.21, *p* = 0.057) in IBD subgroup (Table 2).

**Fig 3:**
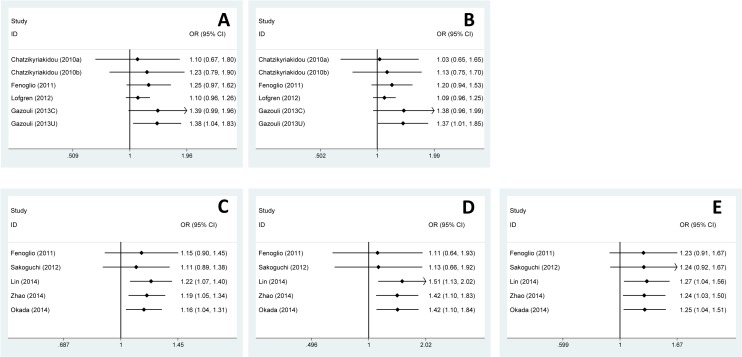
Cumulative meta-analysis of the association between rs2910164 and ADs risk. Every rhombus represents the pooled OR when studies accumulated over time, and the horizontal line represents the 95% CI of the pooled ORs. **(A)** Heterozygote model, GC vs GG, Caucasian subgroup, random model. **(B)** Dominant model, GC+CC vs GG, Caucasian subgroup, random model. **(C)** Allele model, C vs G, Other diseases subgroup, fixed model. **(D)** Homozygote model, CC vs GG, Other diseases subgroup, fixed model. **(E)** Dominant model, GC+CC vs GG, Other diseases subgroup, fixed model.

The cumulative meta-analysis showed that the significance of ORs emerged after the studies published in 2013 enrolled in Caucasian subgroup (Fig 3A and B), while after the studies published in 2014 enrolled in other disease subgroup (Fig 3C, D, and E).

### Evaluation of heterogeneity

The heterogeneities among studies were obvious in the overall comparisons (I^2^ = 77.9%, Tau^2^ = 0.044, *p* = 0.000). The meta-regression analysis was conducted to further explore sources of heterogeneity. We assessed allele comparison by potential sources of publication year, country, genotyping methods, number of genotypes and alleles, and number of female and male in cases. None of the potential sources above could explain the heterogeneity by meta-regression analysis. However, when we compared the frequencies of G allele in controls, we found that the heterogeneity could partly (Adjusted R^2^ = 32.7%) explained by the variation of frequencies of G allele in controls.

### Sensitivity and publication bias analysis

**Fig 4:**
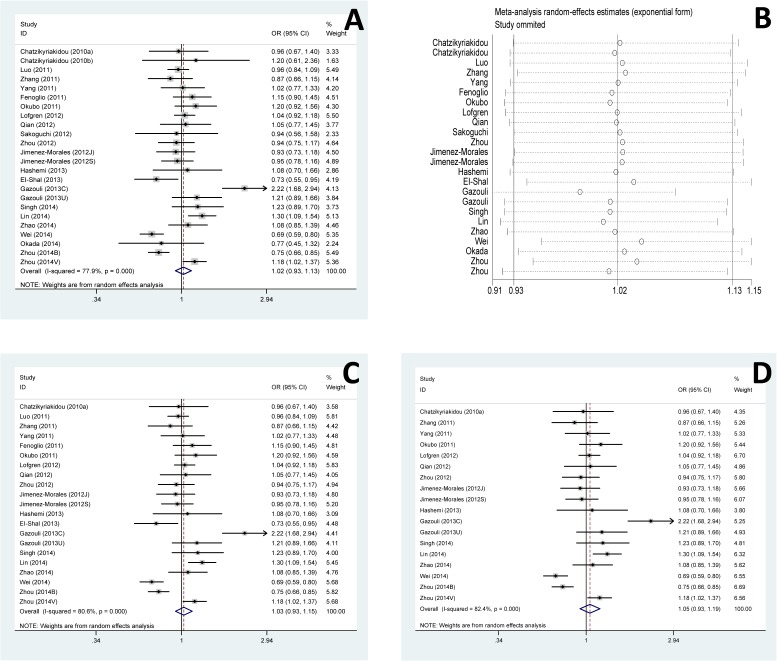
Sensitivity analysis of association of rs2910164 and ADs risk. **(A)** Pooled analysis of association of rs2910164 and ADs risk. Allele model, C vs G. **(B)** Sensitivity analysis by iteratively removing one study at a time. **(C)** Sensitivity analysis by removing three studies with low quality. **(D)** Sensitivity analysis by removing six studies without HWE.

**Fig 5:**
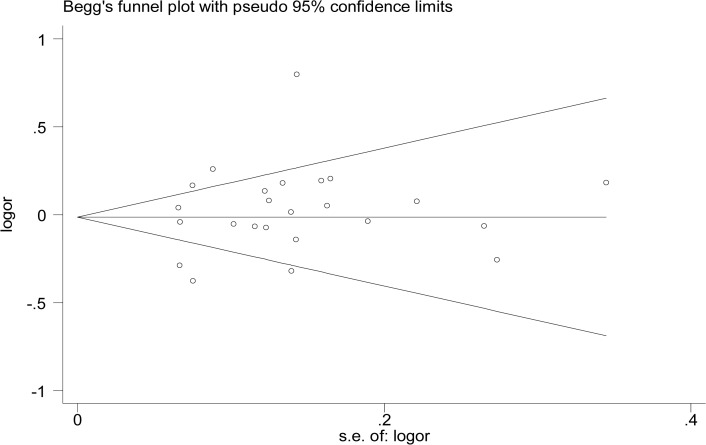
Publication bias on the rs2910164 polymorphism and ADs risk.

We performed the sensitivity analysis to test the influence of a single study on the overall meta-analysis by deleting each study once a time. As a result, the pooled estimate didn’t show significant difference, which indicated that the results in this meta-analyses were statistically reliable. Moreover, the pooled ORs did not vary much even after the three studies [15, 24, 34] with low quality or six studies [15, 16, 19, 24, 27, 34] without HWE removed (Fig 4). No evidence of publication bias was found in current meta-analysis, identified by the Funnel plots, Egger’s test (*p* = 0.261) and Begg’s test (*p* = 0.862) (Fig 5).

## Discussion

Solid evidences had shown that miRNAs played important roles in the regulation of cell differentiation, proliferation, metabolism, apoptosis and tumorigenesis [41]. MiR-146a was one of the first miRNAs identified to be involved in innate immune response, and further demonstrated to be related to several types of cancers [42–44].

One SNP of miR-146a, rs2910164 G>C, was found not only related to cancer, but also related to some kinds of ADs [29]. Accumulating evidences emerged to distinguish if there was any relationship between rs2910164 and susceptibility of ADs. However, the results were inconsistent. A meta-analysis published in 2013 showed that there was not any association between rs2910164 and ADs risk [35]. While they did not include a study published by Luo et al [16] in 2011. Maybe due to there was only frequency data of allele and was not any data of each genotype in Luo’s study, which did not meet the inclusion criteria of this meta-analysis [35]. On the contrary, another meta-analysis published in 2014 did find some association there [36], however, some already published studies [15, 20, 25] prior their search date at April 2013 were not included although they should be. Moreover, the only significances of ORs were found in GG+CC/GC genetic model [36], not in the five well used genetic models as shown in the previous meta-analysis [35]. After that, other researchers published several new case-control studies about this topic in 2013 and 2014 [27, 28, 30–34]. So, it was needed to do an updated meta-analysis which included all the published manuscripts, in order to make a clarified conclusion.

In this meta-analysis, we enrolled 24 studies and pooled the corresponding data including 7591 cases and 9677 controls, and all data of these samples were available in the original publications. Our analysis revealed that there was not any significance of pooled OR. However, in stratified analyses, we found GC genotype and GC+CC genotype were significantly related to the increased susceptibility of ADs in Caucasian subgroup. As in other diseases subgroup, significantly increased risk was observed to be associated with C allele, CC genotype and GC+CC genotype in allele model, homozygote model and dominant model, respectively. All these differences were not found in the two previous meta-analyses [35, 36]. These differences between our data and that in previous meta-analyses may be explained by the different study number and different sample size enrolled in these analyses. As we included more studies and an enlarged sample size, our data should be recognized more powerful.

In cumulative meta-analysis, we found the significance of ORs after new case-control studies published in 2013 and 2014 enrolled. These data demonstrated the correlation of rs2910164 and susceptibility of ADs. In addition, not only sensitivity analysis by iteratively removing one study at a time, but also analysis removing three studies [15, 24, 34] with low quality or six studies [15, 16, 19, 24, 27, 34] without HWE, showed similar and consistent results. Thus, the results of cumulative meta-analysis and sensitivity analysis indicated the robustness of our data. There were still some limitations in our studies. First, although there were 24 studies included, the studies for some stratified analyses were limited. For example, there were only two studies for Latin-American subgroup. Second, there were obvious heterogeneities between different groups for some genetic model. Although the meta-regression and sensitivity analyses were conducted and we found that the variation of G allele frequency in controls could partly explain some heterogeneity, the results still needed to be treated with caution. Third, although there was some significance of ORs in some genetic models, the summary ORs were not very high even after the new studies enrolled in the cumulate meta-analyses. So, future well-designed large studies on this topic will be welcomed to confirm this conclusion. Finally, only rs2910164 in miR-146a was included in this study. However, there were more other SNPs in miR-146a and more other genes could also contribute to susceptibility of ADs. Not only the effect of the SNPs or genes, but also the interaction or network among these genetic locations, should be studied in the future. Furthermore, our data did not study the gene-environment interactions based on the lack of this interaction from the original case-control studies. So, in the future, studies investigating the gene-environment interactions would also help to make clear of the role of the genetic locations in the pathogen of ADs.

## Conclusions

Taken together, our data demonstrated that the miR-146a rs2910164 G>C polymorphism was related to the susceptibility of ADs and our findings should be validated by future well-designed large studies.

## Supporting Information

S1 PRISMA Checklist PRISMA 2009 Checklist. (DOC)

S2 Checklist Meta-analysis on Genetic Association Studies Checklist. (DOCX)

S1 Table Scale for methodological quality assessment. (DOCX)
