# Supplementary material for: Meta-Analysis of MicroRNA-146a rs2910164 G>C Polymorphism Association with Autoimmune Diseases Susceptibility, an Update Based on 24 Studies
Source: PLoS One. 2015 Apr 1;10(4):e0121918. doi: 10.1371/journal.pone.0121918 (PMC4382023; doi:10.1371/journal.pone.0121918)
Supplement: S1 Table — (DOCX) [file pone.0121918.s003.docx]

**Supplementary**

**S1 Table. Scale for methodological quality assessment**

| Criteria | Score |
| --- | --- |
| 1. Representativeness of cases |  |
| Autoimmune diseases (ADs) diagnosed according to acknowledged criteria | 2 |
| Mentioned the diagnosed criteria but not specifically described | 1 |
| Not mentioned | 0 |
| 2. Source of controls |  |
| Population or community based | 3 |
| Hospital-based ADs-free controls | 2 |
| Healthy volunteers without total description | 1 |
| ADs-free controls with related diseases | 0.5 |
| Not described | 0 |
| 3. Sample size |  |
| >300 | 2 |
| 200-300 | 1 |
| <200 | 0 |
| 4. Quality control of genotyping methods |  |
| Repetition of partial/total tested samples with a different method | 2 |
| Repetition of partial/total tested samples with the same method | 1 |
| Not described | 0 |
| 5. Hardy-Weinberg equilibrium (HWE) |  |
| Hardy-Weinberg equilibrium in control subjects | 1 |
| Hardy-Weinberg disequilibrium in control subjects | 0 |
